# Mechanical, Structural and Electronic Properties of CO_2_ Adsorbed Graphitic Carbon Nitride (g-C_3_N_4_) under Biaxial Tensile Strain

**DOI:** 10.3390/ma14154110

**Published:** 2021-07-23

**Authors:** Li-Hua Qu, Chong-Gui Zhong, Peng-Xia Zhou, Jian-Min Zhang

**Affiliations:** 1 School of Science, Nantong University, Nantong 226019, China; chgzhong@ntu.edu.cn (C.-G.Z.); ntzhoupx@ntu.edu.cn (P.-X.Z.); 2 College of Physics and Information Technology, Shaanxi Normal University, Xi’an 710062, China; jmzhang@snnu.edu.cn

**Keywords:** CO_2_, graphitic carbon nitride, strain, first-principles

## Abstract

We investigate mechanical, structural and electronic properties of CO_2_ adsorbed graphitic carbon nitride (g-C_3_N_4_) system under biaxial tensile strain via first-principles calculations. The results show that the stress of CO_2_ adsorbed g-C_3_N_4_ system increases and then decreases linearly with the increasing biaxial strain, reaching maximum at 0.12 strain. This is primarily caused by the plane N–C stretching of the g-C_3_N_4_. Furthermore, both the Perdew-Burke-Ernzerhof (PBE) and Heyd- Scuseria-Ernzerhof screened hybrid functional (HSE06) band gaps show direct-indirect transitions under biaxial tensile strain and have the maximum also at 0.12 strain. It is found that there is large dipole transition matrix element around Γ point, leading high optical absorption coefficients of the deformed adsorption system, which would be of great use for the applications of new elastic nanoelectronic and optoelectronic devices.

## 1. Introduction

The greenhouse effect is the rise in temperature caused by the rapid energy consumption on the global scale. This situation commits us to exploring effective methods for developing renewable energy. One of the most promising methods is to boost photocatalytic CO_2_ reduction into hydrocarbon solar fuels, which can solve energy crisis and simultaneously protect our environment [1,2,3,4]. In 1979 [5], Inoue and his coworkers reported photocatalytic CO_2_ reduction fuels using several semiconductors. After this, wide band gap semiconductor materials for CO_2_ photoreduction have been studied, including CuO, TiO_2_ and NiO and some metal sulfides [6,7,8,9]. All these photocatalysts are usually inorganic semiconductors, which distinguishes from the natural enzymes in plant tissues. Hence it is noted that biomimetic photocatalytic materials for photocatalytic CO_2_ reduction are widely studied [10,11]. As an organic semiconductor photocatalyst, graphitic carbon nitride (g-C_3_N_4_) has attracted much attention due to its visible-light activity, nontoxic resource, low-cost, promising applications in water splitting and what matters more is its appropriate band positions for CO_2_ photoreduction [2,12,13].

Adsorption of CO_2_ on the g-C_3_N_4_ is critical for the photocatalytic activity [14,15]. In addition, the adsorption structure, stability and strength are very important for the electronic behavior. In recent years, the research on CO_2_ photoreduction by g-C_3_N_4_- based photocatalysts has aroused a lot of concern and discussion [2,12,13,14]. For instance, sulfur doped g-C_3_N_4_ are designed to improve photocatalytic CO_2_ reduction performance [15]. g-C_3_N_4_ nanocomposites have been designed for effective CO_2_ photoreduction, since the e−/h+ separation efficiency has risen, including Cu-NPs/g-C_3_N_4_ [14], g-C_3_N_4_/Ag/m-CeO_2_ [16] and Sr_2_Ta_2_O_7_/S-doped g-C_3_N_4_ [17] heterojunctions.

In addition, Caux et al. [18] have studied Pt-g-C_3_N_4_ for CO_2_ photoreduction to improve the catalytic performance ascribed to the charge-separation efficiency. Different from these composite photocatalysts prepared by expensive metal salts, the preparation of pristine g-C_3_N_4_ photocatalyst can be made facilely by thermally polycondensing the cheap N-rich precursors, such as cyanamide, dicyanamide, urea and melamine [19,20]. The outstanding characteristics of g-C_3_N_4_ can be very beneficial in water splitting, CO_2_ photoreduction, catalytic organic synthesis, organic contaminants purification and fuel cells [20]. In previous years, there are several excellent research on the g-C_3_N_4_ preparation and applications [21,22]. In addition, all these studies result from the changes of the local atomic structure, electronic states and mechanical behavior of materials. Furthermore, strain is almost inevitable and the strain effects on the structural and electronic properties of two-dimensional materials have been studied for decades. Sometimes, strain is intentionally applied to improve mobility, as in the strained silicon technology, which is used in modern microelectronics [23]. Thus, it is crucial to understand how the strain affects the further deformation, electronic structures and mechanical properties for the CO_2_ adsorbed g-C_3_N_4_.

In present paper, we investigate the mechanical, structural and electronic properties of biaxial tensile strain tuned CO_2_ adsorbed g-C_3_N_4_ system based on first-principles density functional theory (DFT) [24,25] calculations. The rest of the paper is organized as follows. Section 2 shows the calculation methods and optimized structures of CO_2_ adsorbed g-C_3_N_4_ system. Section 3 is devoted to the effects of biaxial strain regulated mechanical, structural and electronic properties of the adsorption system. Section 4 presents the conclusions.

## 2. Computational Methods

**Figure 1:**
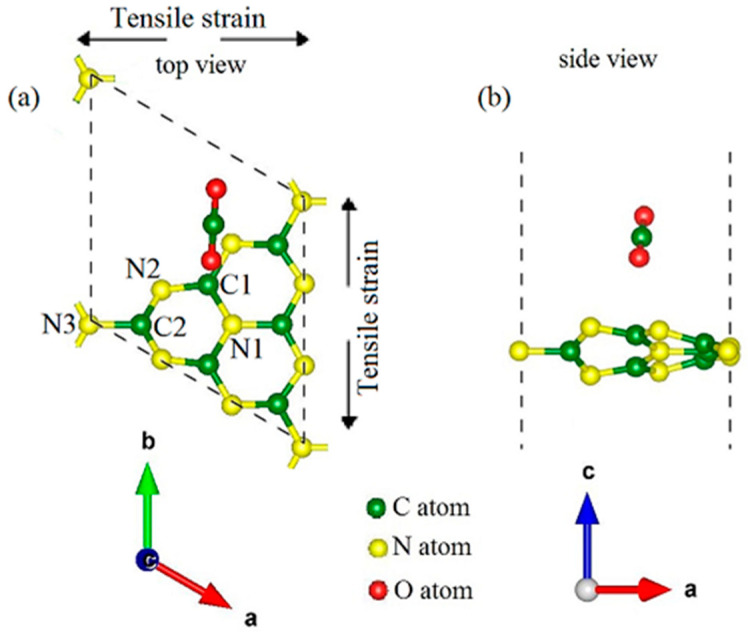
(**a**,**b**) are the top and side views of the optimized CO_2_ adsorbed g-C_3_N_4_ (without strain) system within the unit cell, respectively, which is represented by the dashed black parallelogram. The green, yellow and red balls represent C, N and O atoms, respectively. In addition, the orientation of biaxial tensile strain is signed with black arrows. For the pristine g-C_3_N_4_, there are three types of N and two types of C (N1, N2, N3, C1 and C2).

Our calculations are performed using DFT with the generalized gradient approximation of Perdew-Burke-Ernzerhof (GGA-PBE) form [26], as implemented in the Vienna ab initio Simulation Package (VASP) code [24,25,26,27,28,29]. In addition, the projector augmented wave (PAW) [26] approach is derived. 5 × 5 × 1 Monkhorst-Pack k-point meshes [30] are used to sample the Brillouin zone. The PBE and HSE06 exchange-correlation functionals are adopted. The wave functions are expanded in a plane-wave basis set with an energy cutoff of 450 eV. The total energy and atomic forces are converged to 10^−6^ eV and 0.02 eV/Å. The biaxial strain can be calculated with the following equation [31]:

ε = (a − a_0_)/a_0_ = (b − b_0_)/b (1)

where a, b and a_0_, b_0_ are the deformed (stretched or shrunken) and initial equilibrium lattice constants of unit cell. Figure 1a,b show the top and side views of unit cell for the CO_2_ adsorbed g-C_3_N_4_ system, respectively, which are represented by the dashed black parallelogram. The green, yellow and red balls represent C, N and O atoms, respectively. In addition, the orientation of biaxial tensile strain is marked with black arrows. The unit cell of pristine g-C_3_N_4_ contains three types of N atoms and two types of C atoms (N1, N2, N3, C1 and C2), therefore there are four kinds of N–C bonds (N1–C1, C1–N2, N2–C2 and N3–C2) (shown in Figure 1). The optimized lattice constant of pristine g-C_3_N_4_ turns out to be 7.15 Å, which is consistent with the previous studies [32,33]. We investigate optimization of adsorption system under biaxial tensile strain from 0 to 0.2 with an increment of 0.01 per step. Upon CO_2_ adsorption, the planar g-C_3_N_4_ structure of becomes buckled (puckered). Previous research has shown that CO_2_ prefers to adsorb nearly parallel to the g-C_3_N_4_ plane [34]. In this work, gas-phase calculations have proven useful because they provide in an unperturbed way rather detailed insight into the elementary steps of numerous transformations mediated by the catalysts.

## 3. Results

### 3.1. Mechanical and Structural Properties

**Figure 2:**
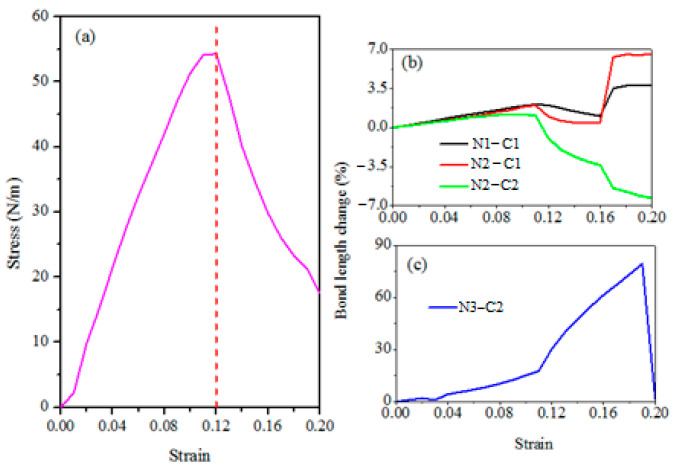
(**a**) the stress-strain curves of the CO_2_ adsorbed g-C_3_N_4_ system. (**b**,**c**) are the N–C bond length change-strain curves.

The stress-strain relations of CO_2_ adsorbed g-C_3_N_4_ system are displayed in Figure 2a. The stress increases almost linearly with the increasing biaxial tensile strain and reaches maximum at about 0.12 strain, then it suddenly drops as the strain increases. The phenomenon of linear elasticity in the CO_2_ adsorbed g-C_3_N_4_ system suggests that its stretchability may not reach high values, because its stretching of bonds have limitations. In order to study the deformation mechanism in more details, we display the bond lengths-strain curves in Figure 2b,c. It has been found that the C–O bond lengths change relatively little with the increasing strain, so we only consider the N–C bonds here. It can be noticed that the outside N3–C2 bond (signed in Figure 1) is stretched more than the inner ones (N1–C1, N2–C1 and N2–C2 in Figure 1), because of the stronger stress concentration around the pore tip. From Figure 2b, we can see that the growth of all the inner bonds (N1–C1, N2–C1 and N2–C2) start to fall at 0.12 strain and show opposite changes of up and down at around 0.16 strain, resulting the decrease of stress-strain curve in Figure 2a. However, the outside N3–C2 bond continues to grow until strain of 0.18, as shown in Figure 2c.

**Figure 3:**
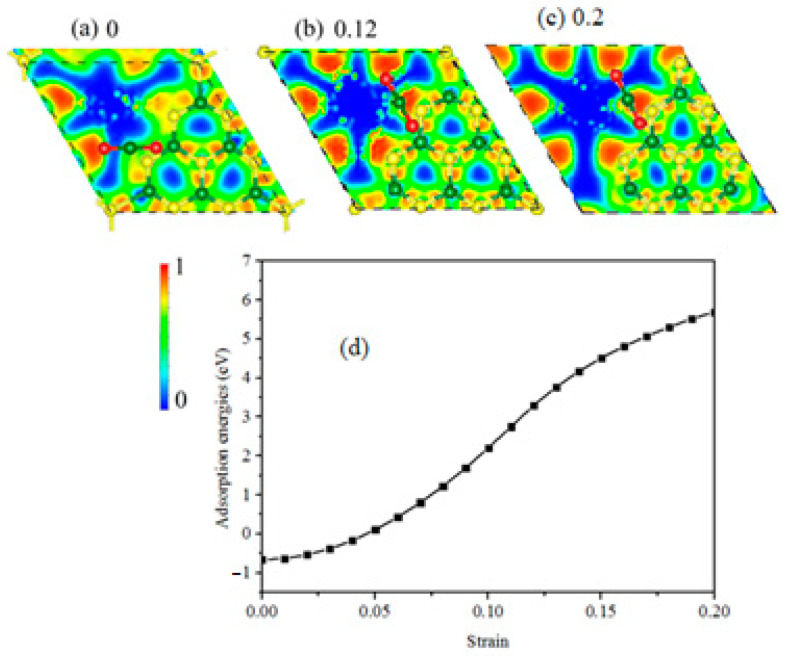
(**a**–**c**) are the atomic structures of the CO_2_ adsorbed g-C_3_N_4_ system at 0, 0.12 and 0.2 strain, respectively. Contours illustrate the electron localization function (ELF) within the unit-cell. (**d**) shows the adsorption energy-strain curve of the CO_2_ adsorbed g-C_3_N_4_ system.

In Figure 3a,b, we draw the CO_2_ adsorbed g-C_3_N_4_ system along with its the electron localization function (ELF) [35] contour at biaxial strain of 0 and 0.12, and the ELF takes a value between 0 and 1. From the representation of the bond in the CO_2_ adsorbed g-C_3_N_4_ system, it can be determined that the outside N3–C2 bond breaks at 0.12 and 0.2 strain. It is clear that the ELF contour agrees with the study of the bond length variations, suggesting that these bonds are very active under biaxial strain, because the ELF values of the inner bonds are about 0.85, showing integrity of covalent bonding. Figure 3c shows the adsorption energy-strain curve to analyze the stability, and the adsorption energy Eads is calculated by the following equation [36]:

(2) $$E_{ads}=E_{total}-E_{g\text{-}C_{3}N_{4}}-E_{CO_{2}}$$

where $E_{total}$, $E_{g\text{-}C_{3}N_{4}}$ and $E_{co_{2}}$ are the total energy of CO_2_ adsorbed g-C_3_N_4_ system, g-C_3_N_4_ and CO_2_, respectively. It is worthwhile to note that the adsorption of CO_2_ on g-C_3_N_4_ is highly exothermic (negative adsorption energy) with strain less than 0.05, suggesting that g-C_3_N_4_ can be an excellent gas sensor. In addition, the adsorption energy increases with the increasing strain, indicating stronger interaction between CO_2_ and g-C_3_N_4_. It is interesting to see that energy-strain curve has a maximum slope at 0.12 strain, which means that there is the biggest deformation. Furthermore, the adsorption energy increases almost quadratically with increasing biaxial strain, showing an elastic behavior rather than a viscous one. This study also demonstrates that the CO_2_ adsorbed g-C_3_N_4_ system without strain is more stable. This is because the tensile strain weakens the bonds of the CO_2_ adsorbed g-C_3_N_4_ system, resulting in higher chemical reactivity. This distinct behavior can be ascribed to the adsorption configuration and charge transfer, which will be systematically discussed below.

### 3.2. Electronic Properties

**Figure 4:**
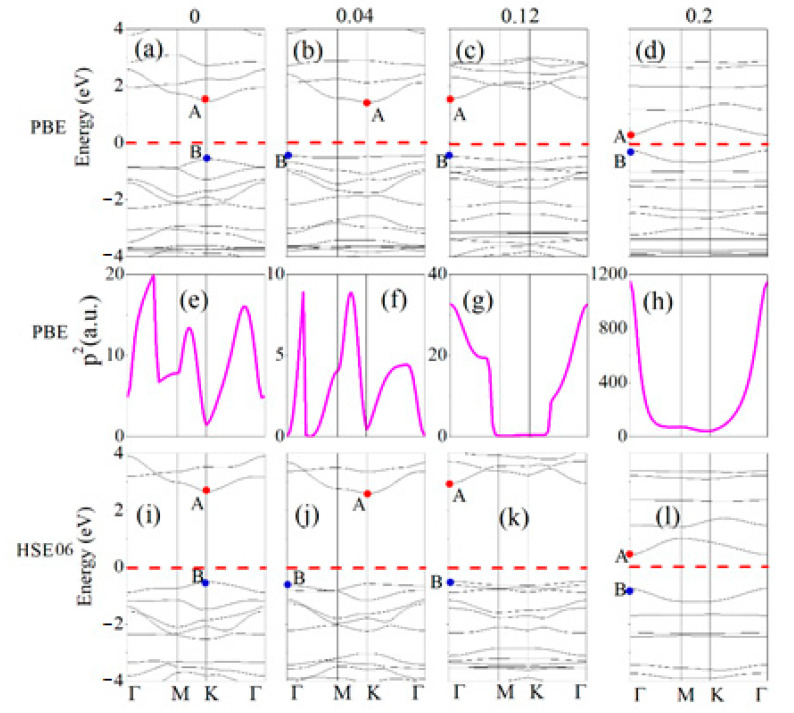
(**a**–**h**) are PBE calculated band structures and transition matrix elements of the CO_2_ adsorbed g-C_3_N_4_ system at 0, 0.04, 0.12 and 0.2 strain. (**i**–**l**) are HSE06 calculated band structures of the CO_2_ adsorbed g-C_3_N_4_ system at 0, 0.04, 0.13 and 0.2 strain. The CBM and VBM band edge positions denoted by the red and blue points, which are signed with A and B, respectively. The Fermi energy is set at zero energy and indicated by the red dashed line.

Band structures calculated with PBE and HSE06 methods for CO_2_ adsorbed g-C_3_N_4_ are shown in Figure 4a–d,i–l. The conduction band minimum (CBM) and valance band maximum (VBM) band edge positions are denoted by the red and blue points, which are signed with A and B, respectively. Apparently, both PBE and HSE06 band gaps exhibit direct (K to K) to indirect (K to Γ) transitions at strain from 0 to 0.04 [see Figure 4a,b,i,j], and then turns to be direct (Γ to Γ) again at 0.12 strain [see Figure 4c,d,k,l]. Moreover, in Figure 4e–h, we plot the PBE high transition probabilities between the topmost valence and the lowest conduction bands, which are revealed by the squares of the dipole transition matrix elements [37], *P*^2^, at all the k-points. It is found that the CO_2_ adsorbed g-C_3_N_4_ system has large dipole transition matrix elements around Γ point, especially at 0.2 strain, indicating strong optical absorption coefficients of photons with energies near the bandgap, which is essential in optoelectronic applications. However, at any strain, the transition matrix amplitudes between band edges are very weak around the M and K points. The rich tunable properties show tremendous potential applications of the biaxial strained CO_2_ adsorbed g-C_3_N_4_ system in elastic nanoelectronic and optoelectronic devices [38,39,40]. Moreover, comparison between the PBE and HSE06 shows that entire shape of band structures is independent of the functional. It is evident that the CBM and VBM are the same in PBE and HSE06. Both functionals show the same type (direct or indirect) of band structures. The band gap is the main difference of the band structures calculated using the two functionals. Therefore, one can qualitatively study the band structures of CO_2_ adsorbed g-C_3_N_4_ with PBE instead of calculating the band in expensive HSE06. In addition, we conducted computational studies (PBE and HSE06) on defected g-C_3_N_4_ to elucidate how the biaxial strain is affected by CO_2_ adsorption in the supporting information Figure S1. It is found that the band structures show metal states under 0 to 0.2 strain.

**Figure 5:**
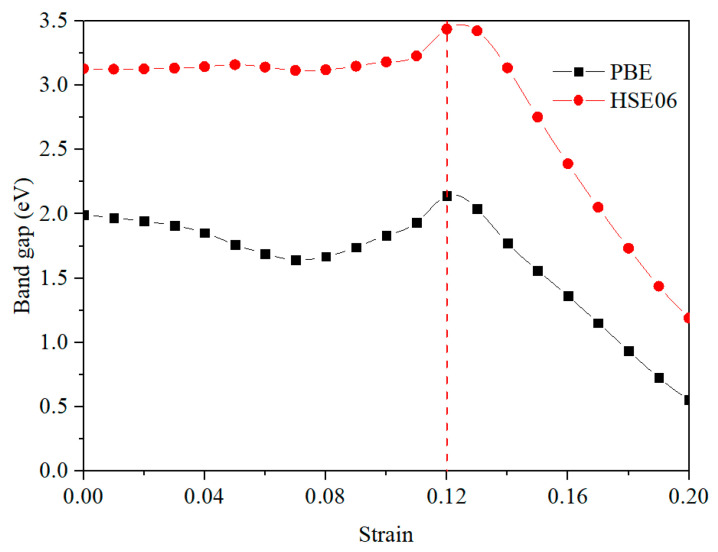
PBE and HSE06 band gap-strain curves of the CO_2_ adsorbed g-C_3_N_4_ system.

Figure 5 shows the PBE and HSE06 band gaps of CO_2_ adsorbed g-C_3_N_4_ as a function of strain. The HSE06 can provide a better prediction of band gap in general. In addition, the HSE06 band gaps are found to be higher than the PBE values under the same strain. Furthermore, the band gap-strain curves of the two methods have the same changing tendency that both methods reach the maximum at 0.12 strain, where the indirect-direct transition occurs, and it is the maximum tensile strength point of the CO_2_ adsorbed g-C_3_N_4_ system.

## 4. Conclusions

In this work, we have investigated mechanical, structural and electronic properties of CO_2_ adsorbed g-C_3_N_4_ system under biaxial tensile strain by performing first-principles calculations. The CO_2_ adsorbed g-C_3_N_4_ system shows linear elasticity and has high strength at 0.12 strain, which is mainly induced by the stretching of N-C bonds in the g-C_3_N_4_ plane. In addition, the outside N-C bonds are stretched more than the inner ones. The unstrained CO_2_ adsorbed g-C_3_N_4_ is more stable because of the bonds weakened by the stretching strain. Both the PBE and HSE06 band gaps exhibit direct- indirect transitions under biaxial tensile strain and reach maximum at 0.12 strain. There are large dipole transition matrix elements around Γ point, showing high optical absorption coefficients, which would be beneficial to the applications of elastic nanoelectronic and optoelectronic devices.

## Data Availability

The data presented in this study are available on request from the corresponding author.

## Supplementary Materials

The following are available online at https://www.mdpi.com/article/10.3390/ma14154110/s1, Figure S1: (a–j) are PBE and HSE06 calculated band structures of the CO_2_ adsorbed on defective g-C_3_N_4_ system at 0, 0.05, 0.1, 0.15 and 0.2 strain. The Fermi energy is set at zero energy and indicated by the red dashed line. (k) is the optimized structure of the CO_2_ adsorbed on defective g-C_3_N_4_ system without strain.

## References

[1] Nam S., Jeong Y.J., Park C.E., Jang J. (2017). Enhanced gas barrier properties of graphene-TiO_2_ nanocomposites on plastic substrates assisted by UV photoreduction of graphene oxide. Org. Electron..

[2] Raganati F., Ammendola P. (2021). Sound-assisted fluidization for temperature swing adsorption and calcium looping: A review. Materials.

[3] Barroso-Bogeat A., Blanco G., Pérez-Sagasti J.J., Escudero C., Pellegrin E., Herrera F.C., Pintado J.M. (2021). Thermocatalytic CO_2_ conversion over a nickel-loaded ceria nanostructured catalyst: A nap-xps study. Materials.

[4] Šupić S., Malešev M., Radonjanin V., Bulatović V., Milović T. (2021). Reactivity and pozzolanic properties of biomass ashes generated by wheat and soybean straw combustion. Materials.

[5] Inoue T., Fujishima A., Konishi S., Honda K. (1979). Photoelectrocatalytic reduction of carbon dioxide in aquous suspensions of semiconductor powders. Nature.

[6] Shekaaria A., Jafaria M. (2020). Unveiling the first post-graphene member of silicon nitrides: A novel 2D material. Comput. Mater. Sci..

[7] Jantarasorn N., Mekasuwandumrong O., Kelly P., Praserthdam P. (2019). Reactive magnetron sputter deposition of copper on TiO_2_ support for photoreduction of CO_2_ to CH_4_. IOP Conf. Ser. Mater. Sci. Eng..

[8] Han C., Zhang R., Ye Y., Wang L., Ma Z., Su F., Xie H., Zhou Y., Wong P.K., Ye L. (2019). Chainmail co-catalyst of NiO shell-encapsulated Ni for improving photocatalytic CO_2_ reduction over g-C_3_N_4_. J. Mater. Chem. A.

[9] Suzuki T.M., Takayama T., Sato S., Iwase A., Kudo A., Morikawa T. (2018). Enhancement of CO_2_ reduction activity under visible light irradiation over Zn-based metal sulfides by combination with Ru-complex catalysts. Appl. Catal. B-Environ..

[10] Tseng I.H., Kang L.H., Chang P.Y., Tsai M.H., Yeh J.M., Yang T.I. (2019). Biomimetic polyimide-supported cuprous oxide photocatalytic film with tunable hydrophobicity, improved thermal stability, and photocatalytic activity toward CO_2_ reduction. ACS Omega.

[11] Li N., Liu J., Liu J.J., Dong L.Z., Xin Z.F., Teng Y.L., Lan Y.Q. (2019). Adenine components in biomimetic metal-organic frameworks for efficient CO_2_ photoconversion. Angew. Chem..

[12] Ma W., Wang N., Guo Y., Yang L., Lv M., Tang X., Li S. (2020). Enhanced photoreduction CO_2_ activity on g-C_3_N_4_: By synergistic effect of nitrogen defective-enriched and porous structure, and mechanism insights. Chem. Eng. J..

[13] Wang H., Li H., Chen Z., Li J., Li X., Huo P., Wang Q. (2020). TiO_2_ modified g-C_3_N_4_ with enhanced photocatalytic CO_2_ reduction performance. Solid State Sci..

[14] Sun Z., Fang W., Zhao L., Wang H. (2020). 3D porous Cu-NPs/g-C_3_N_4_ foam with excellent CO_2_ adsorption and schottky junction effect for photocatalytic CO_2_ reduction. Appl. Surf. Sci..

[15] Gupta B., Gupta A.K., Ghosal P.S., Tiwary C.S. (2020). Photo-induced degradation of bio-toxic Ciprofloxacin using the porous 3D hybrid architecture of an atomically thin sulfur-doped g-C_3_N_4_/ZnO nanosheet. Environ. Res..

[16] Wang H., Guan J., Li J., Li X., Ma C., Huo P., Yan Y. (2020). Fabricated g-C_3_N_4_/Ag/m-CeO_2_ composite photocatalyst for enhanced photoconversion of CO_2_. Appl. Surf. Sci..

[17] Zeng W., Bian Y., Cao S., Zhu A., Qiao L., Ma Y., Tan P., Ma Q., Dong R., Pan J. (2019). Construction of two dimensional Sr_2_Ta_2_O_7_/S-doped g-C_3_N_4_ nanocomposites with Pt cocatalyst for enhanced visible light photocatalytic performance. Appl. Surf. Sci..

[18] Caux M., Fina F., Irvine J.T.S., Idriss H., Howe R. (2017). Impact of the annealing temperature on Pt/g-C_3_N_4_ structure, activity and selectivity between photodegradation and water splitting. Catal. Today.

[19] Yan S.C., Li Z.S., Zou Z.G. (2009). Photodegradation Performance of g-C_3_N_4_ Fabricated by directly heating melamine. Langmuir.

[20] Dong F., Wu L.W., Sun Y.J., Fu M., Wu Z.B., Lee S.C. (2011). Efficient synthesis of polymeric g-C_3_N_4_ layered materials as novel efficient visible light driven photocatalysts. J. Mater. Chem..

[21] Gong Y.G., Li M.M., Wang Y. (2015). Carbon nitride in energy conversion and storage: Recent advances and future prospects. ChemsusChem.

[22] Wang Y., Wang X.C., Antonietti M. (2012). Polymeric graphitic carbon nitride as a heterogeneous organocatalyst: From photochemistry to multipurpose catalysis to sustainable chemistry. Angew. Chem. Int. Ed..

[23] Zhang S., Yan Z., Li Y., Chen Z., Zeng H. (2015). Atomically thin arsenene and antimonene: Semimetal-semiconductor and indirect-direct band-gap transitions. Angew. Chem. Int. Ed..

[24] Kresse G., Hafner J. (1993). Ab initio molecular dynamics for liquid metals. Phys. Rev. B.

[25] Kresse G., Furthmüller J. (1996). Efficient iterative schemes for ab initio total-energy calculations using a plane-wave basis set. Phys. Rev. B.

[26] Perdew J.P., Burke K., Ernzerhof M. (1996). Generalized gradient approximation made simple. Phys. Rev. Lett..

[27] Kresse G., Hafner J. (1994). Ab initio molecular-dynamics simulation of the liquid-metal-amorphous-semiconductor transition in germanium. Phys. Rev. B.

[28] Kresse G., Furthmüller J. (1996). Efficiency of ab-initio total energy calculations for metals and semiconductors using a plane-wave basis set. Comput. Mat. Sci..

[29] Kresse G., Joubert D. (1999). From ultrasoft pseudopotentials to the projector augmented-wave method. Phys. Rev. B.

[30] Monkhorst H.J., Pack J.D. (1976). Special points for Brillonin-zone integrations. Phys. Rev. B.

[31] Qu L.H., Yu J., Mu Y.L., Fu X.L., Zhong C.G., Min Y., Zhou P.X., Zhang J.M., Zou Y.Q., Lu T.S. (2019). Strain tunable structural, mechanical and electronic properties of monolayer tin dioxides and dichalcogenides SnX_2_ (X = O, S, Se, Te). Mater. Res. Bull..

[32] Zhu B., Zhang J., Jiang C., Cheng B., Yu J. (2017). First principle investigation of halogen-doped monolayer g-C_3_N_4_ photocatalyst. Appl. Catal. B Environ..

[33] Cui J., Liang S., Wang X., Zhang J. (2015). First principle modeling of oxygen-doped monolayer graphitic carbon nitride. Mater. Chem. Phys..

[34] Zhu B., Zhang L., Xu D., Cheng B., Yu J. (2017). Adsorption investigation of CO_2_ on g-C_3_N_4_ surface by DFT calculation. J. CO*_2_* Util..

[35] Silvi B., Savin A. (1994). Classification of chemical-bonds based on topological analysis of electron localization functions. Nature.

[36] Ni J., Quintana M., Song S. (2020). Adsorption of small gas molecules on transition metal (Fe, Ni and Co, Cu) doped graphene: A systematic DFT study. Physica E.

[37] Maughan A.E., Ganose A.M., Bordelon M.M., Miller E.M., Scanlon D.O., Neilson J.R. (2016). Defect tolerance to intolerance in the vacancy-ordered double perovskite semiconductors Cs_2_SnI_6_ and Cs_2_TeI_6_. J. Am. Chem. Soc..

[38] Zhou Y., Zhang L., Tao S. (2019). Mesoporous ZrO_2_ Nanopowder catalysts for the synthesis of 5-hydroxymethylfurfural. ACS Appl. Nano Mater..

[39] Bayan S., Gogurla N., Midya A., Singha A., Ray S.K. (2017). Plasmon mediated enhancement and tuning of optical emission properties of two dimensional graphitic carbon nitride nanosheets. Nanotechnology.

[40] Bayan S., Midya A., Gogurla N., Singha A., Ray S.K. (2017). Origin of modified luminescence response in reduced graphitic carbon nitride nanosheets. J. Phys. Chem. C.

