# Supplementary material for: Mechanical, Structural and Electronic Properties of CO2 Adsorbed Graphitic Carbon Nitride (g-C3N4) under Biaxial Tensile Strain
Source: Materials (Basel). 2021 Jul 23;14(15):4110. doi: 10.3390/ma14154110 (PMC8347895; doi:10.3390/ma14154110)
Supplement: Supplementary file 1 [file materials-14-04110-s001.zip › materials-1234057-supplementary.pdf]

materials

Mechanical, Structural and Electronic Properties of CO_2 Adsorbed Graphitic Carbon Nitride (g-C_3N_4) under Biaxial Tensile Strain

Li-Hua Qu [1,*], Chong-Gui Zhong [1], Peng-Xia Zhou [1] and Jian-Min Zhang [2]

1 School of Science, Nantong University, Nantong 226019, China; chgzhong@ntu.edu.cn (C.-G.Z.); ntzhoupx@ntu.edu.cn (P.-X.Z.)
2 College of Physics and Information Technology, Shaanxi Normal University, Xi'an 710062, China; jmzhang@snnu.edu.cn
* Correspondence: qulihua05@163.com

Citation: Qu, L.-H.; Zhong, C.-G.; Zhou, P.-X.; Zhang, J.-M. Mechanical, Structural and Electronic Properties of CO_2 Adsorbed Graphitic Carbon Nitride (g-C_3N_4) under Biaxial Tensile Strain. *Materials* **2021**, *14*, 4110. https://doi.org/10.3390/ma14154110

Academic Editor: Carlos Javier Duran-Valle

Received: 8 May 2021
Accepted: 20 July 2021
Published: 23 July 2021

Publisher's Note: MDPI stays neutral with regard to jurisdictional claims in published maps and institutional affiliations.

Copyright: © 2021 by the authors. Licensee MDPI, Basel, Switzerland. This article is an open access article distributed under the terms and conditions of the Creative Commons Attribution (CC BY) license (http://creativecommons.org/licenses/by/4.0/).

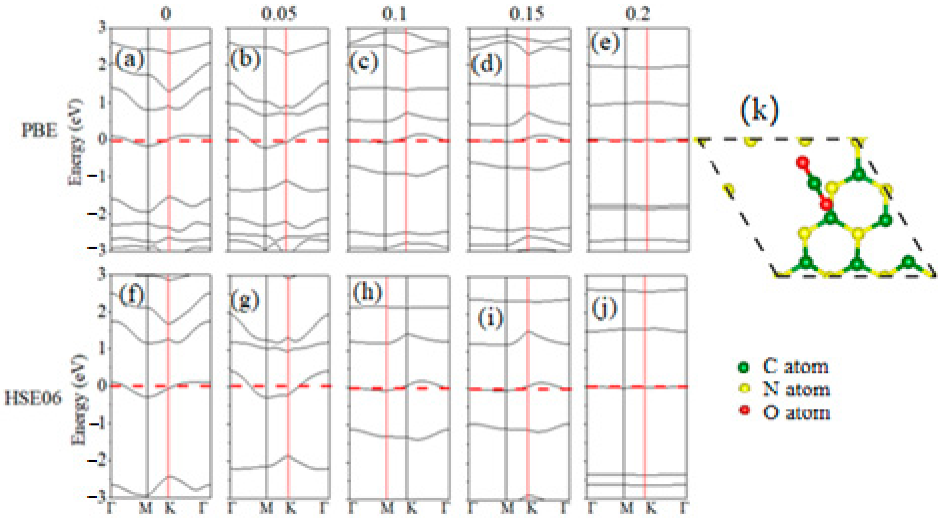

Figure S1 (**a**–**e**) and (**f**–**j**) are PBE and HSE06 calculated band structures of the CO_2 adsorbed on defective g-C_3N_4 system at 0, 0.05, 0.1, 0.15 and 0.2 strain. The Fermi energy is set at zero energy and indicated by the red dashed line. (k) is the optimized structure of the CO_2 adsorbed on defective g-C_3N_4 system without strain.
